# Haemogregarines and Criteria for Identification

**DOI:** 10.3390/ani11010170

**Published:** 2021-01-12

**Authors:** Saleh Al-Quraishy, Fathy Abdel-Ghaffar, Mohamed A. Dkhil, Rewaida Abdel-Gaber

**Affiliations:** 1 Department of Zoology, College of Science, King Saud University, Riyadh 11451, Saudi Arabia; guraishi@yahoo.com (S.A.-Q.); mohameddkhil@yahoo.com (M.A.D.); 2 Zoology Department, Faculty of Science, Cairo University, Cairo 12613, Egypt; fathyghaffar@yahoo.com; 3 Department of Zoology and Entomology, Faculty of Science, Helwan University, Cairo 11795, Egypt

**Keywords:** haemogregarines, gamogony, sporogony, schizongony, molecular analysis

## Abstract

**Simple Summary:**

Taxonomic classification of haemogregarines belonging to Apicomplexa can become difficult when the information about the life cycle stages is not available. Using a self-reporting, we record different haemogregarine species infecting various animal categories and exploring the most systematic features for each life cycle stage. The keystone in the classification of any species of haemogregarines is related to the sporogonic cycle more than other stages of schizogony and gamogony. Molecular approaches are excellent tools that enabled the identification of apicomplexan parasites by clarifying their evolutionary relationships.

**Abstract:**

Apicomplexa is a phylum that includes all parasitic protozoa sharing unique ultrastructural features. Haemogregarines are sophisticated apicomplexan blood parasites with an obligatory heteroxenous life cycle and haplohomophasic alternation of generations. Haemogregarines are common blood parasites of fish, amphibians, lizards, snakes, turtles, tortoises, crocodilians, birds, and mammals. Haemogregarine ultrastructure has been so far examined only for stages from the vertebrate host. PCR-based assays and the sequencing of the *18S rRNA* gene are helpful methods to further characterize this parasite group. The proper classification for the haemogregarine complex is available with the criteria of generic and unique diagnosis of these parasites.

## 1. Introduction

**Figure 1:**
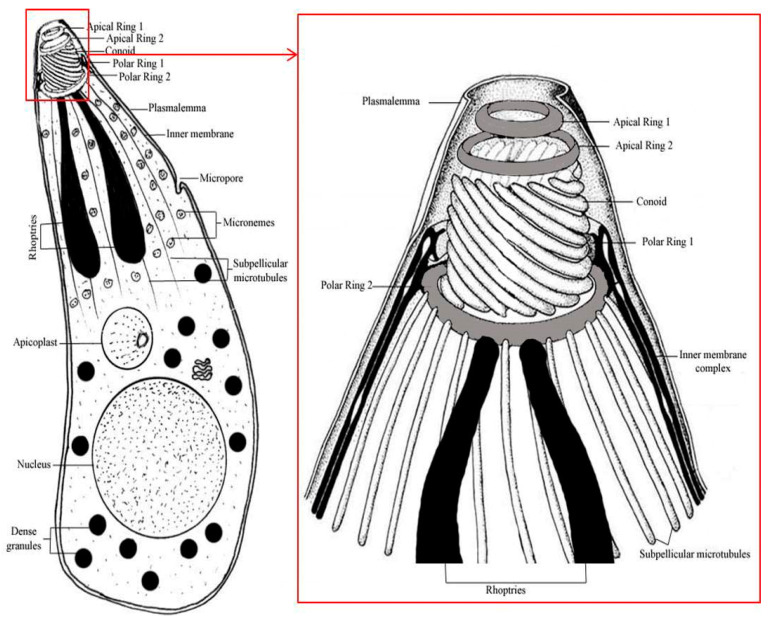
The general structure for the apical complex for Apicomplexa.

**Figure 2:**
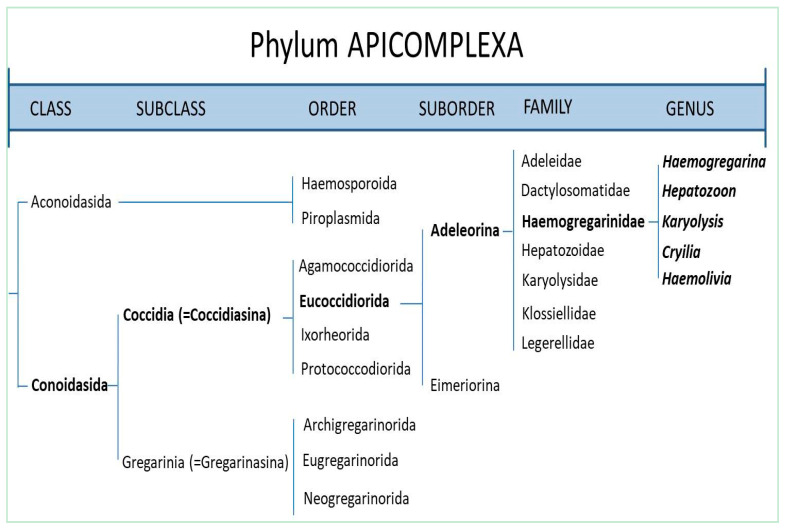
Haemogregarines as a part of phylum Apicomplexa.

**Figure 3:**
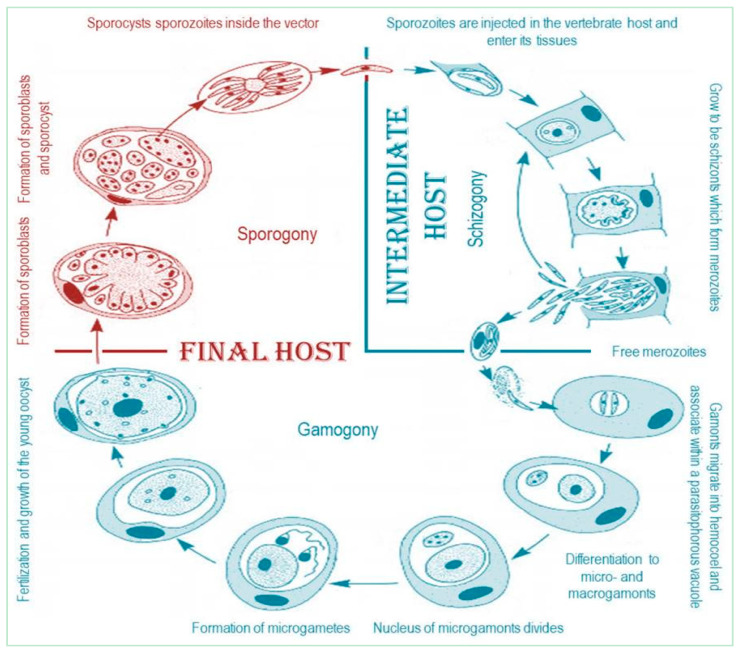
The life cycle of the apicomplexan parasites.

Phylum Apicomplexa was described by Levine [1] to include parasitic protozoa sharing unique ultrastructural features known as the “apical complex” (Figure 1). Haemogregarines (Figure 2) are ubiquitous adeleorine apicomplexan protists inhabiting the blood cells of a variety of ectothermic and some endothermic vertebrates [2,3,4]. They have also an obligatory heteroxenous life cycle (Figure 3), where asexual multiplication occurs in the vertebrate host; while sexual reproduction occurs in the hematophagous invertebrate vector [5]. This family contains four genera, according to Levine [6]: *Haemogregarina* Danilewsky [7], *Karyolysus* Labbé [8], *Hepatozoon* Miller [9], and *Cyrilia* Lainson [10]. Barta [11] conducted a phylogenetic analysis of representative genera in phylum Apicomplexa using biological and morphological features to infer evolutionary relationships in this phylum among the widely recognized groups. The data showed that the biologically diverse Haemogregarinidae family should be divided into at least three families (as suggested by Mohammed and Mansour [12]), were family Haemogregarinidae, containing the genera *Haemogregarina* and *Cyrilia*; family Karyolysidae Wenyon [13], of the genus *Karyolysus*; and family Hepatozoidae Wenyon [13], of the genus *Hepatozoon*, since the four genera currently in the family do not constitute a monophyletic group. The picture is further complicated by evidence from a study by Petit et al. [14] of a new Brazilian toad haemogregarine parasite *Haemolivia stellata*.

It undergoes sporogonic development in its tick host’s gut wall and has a complex life cycle that resembles *Karyolysus* species much more than *Hepatozoon*, *Haemogregarina*, and *Cyrilia* species. Haemogregarines can be morphologically classified based on the developmental details of sporogonic phases of the parasite in the vector, which provide the main characters for classification, the morphology of gametocytes in the red blood cells, and an evaluation of the stages of development [15,16]. Although useful, this methodology is not sufficient for a taxonomic diagnosis [17,18] also the classical systematics has been problematic because of the variability to which morphological details are subjected [19]. Therefore, the use of molecular methods from blood or tissue samples [20,21,22], with appropriate molecular phylogeny study, became an essential adjunct to existing morphological and biological characters for use in the inference of evolutionary history relationships among haemoprotozoan parasites [23,24,25]. Molecular data has been carried out based using PCR assays targeting the nuclear 18s ribosomal RNA gene, which have been extensively applied to characterize hemoparasites DNA more fully in the absence of complete life cycles [26,27,28,29,30,31,32].

In the present critical review of the haemogregarines complex, the proper classification, the criteria of generic and unique diagnosis, and the cosmopolitan distribution of haemogregarines among the vertebrate and invertebrate hosts are examined because of their relevant characteristic and taxonomic revisions.

## 2. Materials and Methods

This review included all related published scientific articles from January 1901 to December 2020. This article was conducted by searching the electronic databases NCBI, ScienceDirect, Saudi digital library, and GenBank database, to check scientific articles and M.Sc./Ph.D. Thesis related to the research topic of this review. Studies published in the English language were only included and otherwise are excluded.

Relevant studies were reviewed through numerous steps. In the first step, target published articles were identified by using general related terms related to the morphological features, such as “Haemogregarines” and “Apicomplex”. The second step involved screening the resulting articles by using highly specific keywords of the generic features for stages in the life cycle of haemogregarines species, including “Merogony”, “Gamogony”, “Sporogony”, “Infective stages”, “Motile stage”, “Infection sites”, and “sporozoites”. The last step of the review focused on selected studies involving the use of molecular analysis for accurate taxonomic identification by using highly specific keywords, including “PCR”, “Genetic markers”, “Variable regions”, “*18S rRNA*”, and “Phylogenetic analysis”.

The obtained data were presented in tables and figures and were: Table 1 representing the characteristic features for the haemogregarines genera, Table 2, Table 3, Table 4, Table 5 and Table 6 showing haemogregarines species, the vertebrate host, site of the merogonic stage, the invertebrate vectors, site of gamogony and sporogonic stages, geographical locality for hosts, and the authors for publishing data, Table 7 with the primer sets used for the amplification and sequencing for the appropriate gene of *18S rRNA* for haemogregarines, and Table 8 representing all the sequenced and deposited haemogregarines in the GenBank database until now.

## 3. Results and Discussion

In this review, the different stages of the apicomplexan life cycle were used to identify haemogregarines. However, in most cases, their assignment to one or another genus cannot be considered more than provisional. Accordingly, about 82 haemogregarines in 155 research articles were identified previously. Osimani [33] stated that the differences between the haemogregarines relied more on the host’s identity than the parasite’s characteristics. Mohammed and Mansour [12] reported that haemogregarines gamonts morphology does not provide generic identification with a reliable key. However, Telford et al. [34], and Herbert et al. [35] stated that the determination of generic haemogregarines should not be based exclusively on the gamonts’ form, the type of parasitized host cells, and their effect on the host and site merogony in host cells. While the most characteristic feature for the basic identification via the sporogonic stage.

The reviewed species belonged to the four genera within Hemogregarinidae (Table 1). Following the parsimony analysis in the phylogenetic study of the representative genera in phylum Apicomplexa performed by Siddall and Desser [36] primarily based on ultrastructural observations, it was concluded that the variations between the different haemogregarines genera are mainly reflected by the sporogony features. Besides, Dvořáková et al. [37] added that the host specificity, together with the haemogregarine’s careful morphological and biological analysis, is a sound criterion for accurate identification. These species are common in different animals as fish (Table 2), amphibians (Table 3), reptiles (Table 4, Table 5, Table 6 and Table 7), birds (Table 8), and mammals (Table 9).

In the schizogony (merogony) stage, haemogregarines are characterized by their considerable ability to invade and develop within different organs and cell types inside the vertebrate host (Table 2, Table 3, Table 4, Table 5, Table 6, Table 7, Table 8 and Table 9). Bray [127] proposed that haemogregarines with schizonts in the liver should be placed in the genus *Hepatozoon*. In contrast, those species that precede schizogony in other organs should belong to another genus as *Haemogregarina* or *Karyolysus*. However, only in the lung of the river turtle, *Trionyx gangeticus* infected with *Haemogregarina gangetica*, was described by Misra [87]. In addition to the usual location of merogonic development in the liver, lung, and spleen, Ball et al. [71] have found certain merogonic stages in the highly infected snakes’ brain and heart. Siddall and Desser [84] described merogonic stages in the lacunar endothelial cells of the circulatory system of the leech and its proboscis, besides the liver, lung, and spleen in the turtle. Yanai et al. [128] also described nodular lesions containing schizonts and merozoites of *Hepatozoon* sp. of the heart’s martens, perisplenic, and perirenal adipose tissues, the diaphragm, mesentery, and tongue. Úngari et al. [102] reported that the genus *Haemogregarina* underwent schizogony in the circulating blood cells as in turtles and fish, and the genus *Hepatozoon* underwent schizogony in the liver. Additionally, there are two morphologically different meronts were the micro- and macromeronts. The presence of these two forms of meronts was mentioned to be a fundamental feature of the whole haemogregarine [74,129,130].

Gametocytes are usually the only stages of the parasite detected by scientists. Their morphology, unfortunately, does not provide a reliable clue to the generic differentiation. Together with other relevant data, their morphological characteristics offer a reliable basis for specific identification [35,67]. The haemogregarines gametocytes appeared as sausage-shaped and generally lie singly within erythrocytes (Table 2, Table 3, Table 4, Table 5, Table 6, Table 7, Table 8 and Table 9), but sometimes free in extracellular space, which is consistent with Telford et al. [34], Sloboda et al. [79] as the presence of free extracellular gametocytes. They are also observed in the leucocytes of fish (Table 2), birds (Table 8), and mammals (Table 9).

The shape, size, and structure of infected blood-corpuscles often undergo considerable changes. Hypertrophy may result directly from the gametocyte’s added intraerythrocytic volume or represent an erythrocyte adaptation to the gametocyte’s presence [53,82,131,132]. An entirely different cell response occurred when the gametocytes of *Hemogregarina* sp. invaded erythrocytes of *Rana berlandieri*. The erythrocytes undergo hypertrophy, and the plasmalemma of the infected erythrocyte demonstrated numerous microvilli-like out-growings. Hussein [133] also described the hypertrophy of *Karyolysus*-infected erythrocytes. Most haemogregarine gametocytes do not invade the host cell’s nucleus but instead move it to the opposite side or the other host cell’s other pole. This is contrary to the effect of the genus *Karyolysus* on the infected erythrocytes. *Karyolysus* has a karyolytic impact on the host cell’s nucleus and is therefore identified *Karyolysus* Reichenow [134].

Little work had been done to identify the actual arthropod vectors of haemogregarines, as the transmission by inoculation of blood was rarely successful. In general, the invertebrate vectors of haemogregarines were the most challenging problem facing this group’s research progress [49]. The haemogregarines displayed a wide distribution of vertebrate host infections, and a large number of invertebrate vectors (Table 2, Table 3, Table 4, Table 5, Table 6, Table 7, Table 8 and Table 9). In all haemogregarines, fertilization is of Adelea type; both micro- and macrogamonts lie in syzygy within the same parasitophorous vacuole. Syzygy can stimulate the production of the associated gamonts in haemogregarines, since only the parasites found in pairs were mostly differentiated, which is consistent with Davies and Smit [42]. Regarding the number of microgametes produced by each microgamont, the members of the suborder Adeleidea were characterized by the production of only a few (four or less) microgametes [135]. Simultaneously, the formation of multiple microgametes has been identified in most haemogregarines species [52]. However, there are some suggestions that multiple microgamete formation does not occur in the entire genus *Hepatozoon* [111]. Regarding the number of flagella in microgametes in haemogregarines, contradictions were recorded. While monoflagellated microgametes have been described for haemogregarines species [74], biflagellated microgametes were also recorded for other haemogregarines [52]. On the other hand, Michel [85] reported non-flagellated microgametes in *Hepatozoon mauritanicum*.

Fertilization follows, leading to the formation of a zygote that becomes an oocyst. The oocyst is surrounded by a flexible membrane rather than a wall, and it produces sporozoites that may undergo further merogony. Sporogony is elucidated for just a few known haemogregarines species, the vast majority of which is supposed to investigate this aspect of their life-cycle, as reported by Forlano et al. [113]. There is also another potential criterion for distinguishing between *Hepatozoon* and *Haemogregarina* based on the presence or absence of oocysts containing sporocysts in the invertebrate vector, which is consistent with Levine [6]. When the developing mite reaches the nymphal stage, the sporozoites attain their maturity. The sporozoites eventually get the nymph’s stomach and pass out with their faeces, which are considered infection sources of the vertebrate host (lizard). The morphological characteristics of the gamonts and meronts found in the blood cells sometimes provide inadequate information for differential diagnoses [37], meaning that assigning species of haemogregarines to one of these genera must be based on the characteristics of its sporogony in the invertebrate vectors [6,64]. However, data on invertebrate vectors and sporogony are missing for the majority of species [23].

Until now, the current taxonomy of haemogregarines is facing a great challenge due to the high variation in gamont morphology, low host specificity, unknown invertebrate hosts in many cases, and fewer details of sporogony. Therefore, molecular approaches are now available to distinguish populations of morphologically identical but genetically different parasites, including DNA and polymerase chain reaction (PCR) based approaches [22,136,137,138,139,140,141]. Some studies based on PCR-based assays as the reference diagnostic test for epidemiological studies, which given their greater sensitivity, particularly for testing different hosts with intermittent levels of parasitemia via a low infection rate by gamonts, as Otranto et al. [114], Haklová-Kočíková et al. [18], Jòzsef et al. [24], Ramos et al. [116], and Mitkova et al. [120]. Notably, all the molecular evidence comes from the complete and partial sequences of the small subunit (SSU) ribosomal DNA (rDNA) 18S gene is a sufficient phylogenetic marker to approximate ordinal level relationships and those within orders [68,98,119,142,143,144,145]. Previous molecular studies of Harris et al. [22] and Barta et al. [19] demonstrated that the haemogregarine species are clustered in sister clades with interspecies linked more with the host geographic distribution, rather than host species. There are universal primer sets that were able to molecularly characterize haemogregarines, as mentioned in Table 10. However, many species with sequences deposited in the GenBank database are not identified correctly at the generic level. Table 11 expressed only haemogregarines identified at the species level and others identified at the generic level are excluded.

## 4. Conclusions

Few haemogregarine characteristics provide a reliable basis for the related parasite to recognized genera. Details of the sporogonic cycle seem to be the only reliable criterion as they are the “Key-stone” in the classification system. Morphological characteristics of the gametocytes do not help in this respect. Features of the schizogonic stages, when these are known, are not much better as criteria of generic value. Molecular phylogenetic studies using the appropriate genetic markers are helpful tools for the accurate taxonomic identification for haemogregarines. Further studies are recommended to include other nuclear and mitochondrial genes to provide more information about the genetic variability among haemogregarines.

## Figures and Tables

**Table 1:** Characters of different groups of haemogregarines used in the parsimony analysis carried out by Barta [19] and Siddall and Desser [36].

Comparable Features	*Karyolysis*	*Haemogregarina*	*Cryilia*	*Hepatozoon*	*Haemolivia*
Conoid present	In all non-gametes	In all non-gametes	In all non-gametes	In all non-gametes	In all non-gametes
Crystalloid bodies +/-	?	+	?	+	+ (fragmented)
Merogeny +/-	+ Intra-cellular	+ Intra-cellular	+ Intra-cellular	+ Intra-cellular	+ Intra-cellular
Micropores +/-	+	+	+	+	+
Mitochondria.	Cristate	Cristate	Cristate	Cristate	Cristate
Mitosis	Centriolar	Centriolar	?	Centriolar	Centriolar
Amylopectin granules +/-	+	-	-	+	+
Polar ring complex +/-	+	+	+	+	+
Gametogenesis	Extra-cellular	Extra-cellular	Extra-cellular	Extra-cellular	Intra-cellular
No. of microgametes/each microgamont	2	4	4	4	2–4
Gamonts	Anisogamous	Anisogamous	Anisogamous	Anisogamous	Anisogamous
Syzygy	+	+	+	+	+
Zygote	Non-motile	Non-motile	Non-motile	Non-motile	Non-motile
Sporogony	Extra-cellular	Extra-cellular	Extra-cellular	Extra-cellular	Intra-cellular
Persistent cysts +/-	-	-	-	+	+
No. of flagella/microgametes	1	1	Absent	1	?
Arrangement of flagella in microgametes	Terminal	Terminal	?	Terminal	Terminal
No. of sporozoites/oocyst	20–30	8	>20	4–16	10–25

Note: (+) presence, (-) absence, (?) not detected.

**Table 2:** Haemogregarines of fish.

Species of Haemogregarines	The Vertebrate Host	Site of Merogony	Invertebrate Vector	Site of Gamogony and Sporogony	Locality	Authors
*Cyrilia gomesi*	*Synbranchus marmoratus*	Leucocytes	*Haementeria lutzi*	Stomach	Sao Paulo, Brazil	Nakamoto et al. [38]
*Haemogregarina bigemina*	*Lipophrys folis* and *Coryphoblrnnius galerita*	Blood cells	*Gnathia maxillaris*	Hindgut	Portugal Atlantic west coast	Davies et al. [39]
*Haemogregarina vltavensis*	*Perca fluviatilis*	Intra-erythrocytic gamonts are only described	--	--	Czechoslovakia	Lom et al. [40]
*Haemogregarina leptocotti*	*Leptocottus armatus*	Blood cells	--	--	California USA	Hill and Hendrickson [41]
*Haemogregarina roelofsi*	*Sebastes melanops*	Blood cells	--	--	California USA	Hill and Hendrickson [41]
*Haemogregarina bigemina*	*Clinus superciliosus* and *Clinus cottoides*	Intra-erythrocytic	*Gnathia africana*	--	South Africa	Davies and Smit [42]
*Haemogregarine* sp.	*Scomber scombrus* L.	Leucocytes	--	--	Northwest and Northeast Atlantic ocean	Maclean and Davies [43]
*Haemogregarina curvata*	*Clinus cottoides*, *Parablennius cornutus*	Intra-erythrocytic	*Zeylanicobdella arugamensis*	Host gut tissue	South Africa	Hayes et al. [44]
*Haemogregarina balistapi*	*Rhinecanthus aculeatus*	Intra-erythrocytic	*Gnathia aureamaculosa*	Host gut tissue	Great Barrier Reef, Australia	Curtis et al. [45]
*Cyrilia* sp.	*Potamotrygon wallacei*	Intra-erythrocytic	*--*	--	Rio Negri	Oliveira et al. [46]
*Haemogregarina daviesensis*	*Lepidosiren paradoxa*	Intra-erythrocytic	*--*	--	Eastern Amazon region	Esteves-Silva et al. [47]

**Table 3:** Haemogregarines of amphibians.

Species of Haemogregarines	The Vertebrate Host	Site of Merogony	Invertebrate Vector	Site of Gamogony and Sporogony	Locality	Authors
*Pseudohaemogregarina nutti*	*Rana nutti*	Erythrocytes and liver	--	--	Germany	Awerenzew [48]
*Haemogregarina theileri*	*Rana angloensis*	Erythrocytes and liver	--	--	Njoro, Kenya	Ball [49]
*Haemolivia stellate*	Brazilian toads	Liver	Ticks	Gut wall	Brazil	Petit et al. [14]
*Haemogregarina nucleobisecans*	*Bufo himalayanus*	Erythrocytes and liver	--	--	India	Ray [50]
*Hepatozoon sipedon*	*Nerodia sipedon* and *Rana pipiens*	Various internal organs	*Culex pipiens* and *Culex territans*	Hemocoel	Ontario, Canada	Smith et al. [51]
*Hepatozoon catesbianae*	*Rana catesbeiana*	Erythrocytes and liver	*Culex territans*	Malpighian tubules	Ontario, Canada	Desser et al. [52]
*Hepatozoon caimani*	*Rana catesbeiana*	Intra-erythrocytic	*Culex fatigans*	Extra-erythrocytic gametocytes	State of Mato Grosso	Lainson et al. [53]
*Hepatozoon theileri*	*Amietia quecketti*	Intra-erythrocytic gamonts are only described	--	--	South Africa	Conradie et al. [54]
*Hepatozoon involucrum*	*Hyperolius marmoratus*	Intra-erythrocytic	--	--	KwaZulu-Natal, South Africa	Netherlands et al. [55]
*Hepatozoon tenuis*	*Afrixalus fornasinii*	Intra-erythrocytic	--	--	KwaZulu-Natal, South Africa	Netherlands et al. [55]
*Hepatozoon thori*	*Hyperolius marmoratus*	Intra-erythrocytic	--	--	KwaZulu-Natal, South Africa	Netherlands et al. [55]

**Table 4:** Haemogregarines of lizards.

Species of Haemogregarines	The Vertebrate Host	Site of Merogony	Invertebrate Vector	Site of Gamogony and Sporogony	Locality	Authors
*Hepatozoon mesnili*	*Gecko verticillatus*	Endothelial cells of all host organs	*Culex fatigans* and *Aedes albopictus*	Stomach	Saigon	Robin [56]
*Haemogregarina triatomae*	*Tupinambis teguixin*	Liver and lung	*Triatoma subrovaria*	Intestine	South America	Osimani [33]
*Hepatozoon argantis*	*Agama mossambica*	Liver	*Argas brumpti*	Gut and homocoelomic cavity	East Africa, Mossambic	Garnham [57]
*Hepatozoon sauromali*	*Sauromalus* sp.	Liver	*Ophionyssus* sp.	Hemocoel	--	Lewis and Wagner [58]
*Haemogregarina* sp.	*Tarentola annularis*	Lung	--	--	Sudan	Elwasila [59]
*Hepatozoon lygosomarum*	*Leiolopisma nigriplantare*	Liver and spleen	*Ophionissus saurarum*	Wall of the gut caeca	Canterbury, New Zealand	Allison and Desser [60]
*Haemogregarina waltairensis*	*Calotes versicolor*	Peripheral blood, liver, lung, and bone marrow	--	--	India	Saratchandra [61]
*Hepatozoon gracilis*	*Mabuya quinquetaeniata*	Liver	*Culex pipienis molesus*	Hemocoel	Giza, Egypt	Bashtar et al. [62]
*Haemogregarina* sp.	*Podarcis bocagei* and *Podarcis carbonelli*	Intra-erythrocytic	--	--	NW Portugal	Roca and Galdón [63]
*Haemogregarina ramadani*	*Acanthodactylus boskianus*	Intra-erythrocytic	--	--	Giza, Egypt	Abdel-Baki and Al-Quraishy [64]
*Hepatozoon* sp.	*Podarcis vaucheri*	Intra-erythrocytic	--	--	Oukaimeden	Moreira et al. [65]
*Haemogregarina* sp.	*Tarentola annularis*	Intra-erythrocytic	*--*	--	Qena, Egypt	Rabie and Hussein [66]
*Karyolysus lacazei* *Karyolysus* sp. *Karyolysus latus*	*Lacerta agilis* *Zootoca vivipara* *Podarcis muralis*	Intra-erythrocytic	*Ophionyssus saurarum* and *Ixodes ricinus*		Poland, Slovakia	Haklová-Kočíková et al. [18]
*Karyolysus paradoxa*	*Varanus albigularis*, *Varanus niloticus*	Intra-erythrocytic	--	--	Ndumo Game Reserve, South Africa	Cook et al. [31]
*Haemogregarina daviesensis*	*Lepidosiren paradoxa*	Intra-erythrocytic	--	--	Eastern Amazon region	Esteves-Silva et al. [47]
*Haemogregarina* sp.	*Scincus scincus*	Intra-erythrocytic	--	--	South Sinai, Egypt	Abou Shafeey et al. [67]
*Karyolysus lacazei*	*Lacerta schreiberi*	Intra-erythrocytic	*Ixodes ricinus*	--	Czech Republic	Zechmeisterová et al. [68]

**Table 5:** Haemogregarines of snakes.

Species of Haemogregarines	The Vertebrate Host	Site of Merogony	Invertebrate Vector	Site of Gamogony and Sporogony	Locality	Authors
*Hepatozoon rarefaciens*	*Drymachon corais*	Lung	*Culex tarsalis, Anopheles albintarus, Aedes sierrensis*	Hemocoel	California, USA	Ball and Oda [69]
*Haemogregarnia* *matruhensis*	*Psammophis schokari*	Intra-erythrocytic	--	--	Egypt	Ramadan [70]
*Hepatozoon fusifex*	*Boa constrictor*	Lung	*Culex tarsalis*	Hemocoel	USA	Ball et al. [71]
*Hepatozoon aegypti*	*Spalerosophis diadema*	Lung	*Culex pipiens molestus*	Hemocoel	Egypt	Bashtar et al. [72]
*Hepatozoon mocassini*	*Agkistrodon piscivorus leucostoma*	Liver parenchyma cells	*Aedes aegypti*	Hemocoel	Louisiana, USA	Lowichik et al. [73]
*Hepatozoon seurati*	*Cerastes cerastes*	Liver, lung, and spleen	*Culex pipiens molestus*	Hemocoel	Aswan, Egypt	Abdel-Ghaffar et al. [74]
*Hepatozoon mehlhorni*	*Echis carinatus*	Liver, lung, and spleen	*Culex pipiens molestus*	Hemocoel	Siwah and Baharia Oasis, Egypt	Bashtar et al. [75]
*Hepatozoon matruhensis*	*Psammophis schokari*	Liver and lung	*Culex pipiens molestus*	Hemocoel	Faiyum, Ismailia, Egypt	Bashtar et al. [76]
*Hepatozoon ghaffari*	*Cerastes vipera*	Liver, lung, and spleen	*Culex pipiens molestus*	Hemocoel	Aswan, Egypt	Shazly et al. [77]
*Hepatozoon sipedon*	*Nerodia sipedon* and *Rana pipiens*	Liver and internal organs	*Culex pipiens*, and *Culex territans*	Hemocoel	Ontario, Canada	Smith et al. [51]
*Haemogregarnia garnhami*	*Psammophis schokari*	Intra-erythrocytic	--	--	Egypt	Saoud et al. [78]
*Hepatozoon ayorgbor*	*Python regius*	Intra-erythrocytic	--	--	Ghana	Sloboda et al. [79]
*Haemogregarnia* sp.	*Cerastes cerastes gasperetti*	Intra-erythrocytic	--	--	Jizan, Saudi Arabia	Al-Farraj [80]
*Hepatozoon garnhami*	*Psammophis schokari*	Intra-erythrocytic	--	--	Riyadh, Saudi Arabia	Abdel-Baki et al. [29]
*Hepatozoon* sp.	*Zamenis longissimus*	Intra-erythrocytic	--	--	Iran	Sajjadi and Javanbakht [81]
*Hepatozoon aegypti*	*Spalerosophis diadema*	Intra-erythrocytic	--	--	Riyadh, Saudi Arabia	Abdel-Haleem et al. [82]

**Table 6:** Haemogregarines of turtles and tortoises.

Species of Haemogregarines	The Vertebrate Host	Site of Merogony	Invertebrate Vector	Site of Gamogony and Sporogony	Locality	Authors
*Hemogregarina nicoriae*	*Nicoria trijuga*	Circulating blood and lung	*Ozobranchus shipleyi*	Intestinal epithelium	Ceylon	Robertson [83]
*Haemogregarina balli*	*Chelydra serpentine serpentina*	Lacunar endothelial cells, liver, lung, and spleen	*Placobdella ornata*	Gastric and intestinal caeca	Ontario, Canada	Siddall and Desser [84]
*Hepatozoon mauritanicum*	*Testudo graeca*	Endothelial cells of all host organs as liver, lung, spleen … etc	*Hyalomma aegyptium*	The intestinal epithelium of the tick	--	Michel [85]
*Haemogregarina pseudomydis*	*Pseudemys scripta elegans*	Leucocytes and Erythrocytes	*Placobdella parasitica*	The intestinal epithelium of the leech	Louisiana, USA	Acholonu [86]
*Haemogregarina gangetica (=H. simondi)*	*Trionyx gangeticus*	Erythrocytes and lung	--	--	India	Misra [87]
*Haemogregarina ganapatii*	*Lissemys punctata granosa*	Peripheral blood and Liver and lung	--	--	India	Saratchandra [61]
*Haemogregarina sinensis*	*Trionyx sinensis*	Erythrocytes and Kupffer’s cells of the liver	*Mooreotorix cotylifer*	Gastric and intestinal caeca of the leech	China	Chai and Chen [88]
*Haemogregarina sp.*	*Emys orbicularis*	Intra-erythrocytic	*Placobdella costata*	--	Romania	Mihalca et al. [89]
*Haemolivia mauritanica*	*Testudo graeca*	Intra-erythrocytic	*Hyalomna aegyptium*	Gut cells	Israel	Paperna [90]
*Haemolivia mauritanica*	Tortoises	Intra-erythrocytic	*Hyalomma aegyptium*	--	Western Palaearctic realm	Široký et al. [91]
*Haemogregarina macrochelysi*	*Macrochelys temminckii*	Intra-erythrocytic	*Leech*	--	Georgia and Florida	Telford et al. [92]
*Haemogregarina stepanowi*	*Emys orbicularis, Mauremys caspica, M. rivulata, M. leprosa*	Intra-erythrocytic	*--*	--	Western Palaearctic	Dvořáková et al. [23]
*Haemogregarina sp.*	*Lissemys punctata* and *Geoclemys hamiltonii*	Intra-erythrocytic	--	--	West Bengal, India	Hossen et al. [4]
*Haemolivia mauritanica*	*Testudo graeca* and *Testudo marginata*	Intra-erythrocytic		--	North African	Harris et al. [93]
*Haemogregarina sp.*	*Rhinoclemmys funera* and *Kinosternon leucostomum*	Intra-erythrocytic	--	--	Costa Rica	Rossow et al. [94]
*Haemogregarina sp.*	*Podocnemis unifilis*	Intra-erythrocytic	--	--	Brazilian Amazonia	Soares et al. [95]
*Haemogregarina sundarbanensis*	*Lissemys punctata*	Intra-erythrocytic	--	--	West Bengal, India	Molla et al. [96]
*Haemogregarina stepanowi*	*Emys orbicularis*	Intra-erythrocytic	--	--	Belgrade Zoo	Jòzsef et al. [24]
*Haemogregarina sp.*	*Podocnemis expansa*	Intra-erythrocytic	--	--	Araguaia River Basin, Brazil	Picelli et al. [97]
*Haemogregarina sacaliae* *Haemogregarina pellegrini*	*Cuora galbinifrons, Leucocephalon yuwonoi, Malayemys subtrijuga, Platysternon megacephalum,*	Intra-erythrocytic	--	--	Southeast Asia	Dvořáková et al. [37]
*Haemogregarina fitzsimonsi* *Haemogregarina parvula*	Land tortorise, *Stigmochelys pardalis*	Intra-erythrocytic	--	--	South African	Cook et al. [31]
*Haemogregarina stepanowi*	*Emys trinacris*	Intra-erythrocytic	--	--	Sicily	Arizza et al. [98]
*Haemogregarina sp.*	*Mauremys caspica*	Intra-erythrocytic	--	--	Iran	Rakhshandehroo et al. [99]
*Haemogregarina sp.*	*Macrochelys temminckii*	Intra-erythrocytic	--	--	Caldwell Zoo, Texas	Alhaboubi et al. [100]
*Haemogregarina sp.*	*Mesoclemmys vanderhaegei*	Intra-erythrocytic	--	--	Brazil	Goes et al. [101]
*Haemogregarina podocnemis*	*Podocnemis Unifilis*	Intra-erythrocytic	--	--	Brazil	Úngari et al. [102]

**Table 7:** Haemogregarines of crocodilians.

Species of Haemogregarines	The Vertebrate Host	Site of Merogony	Invertebrate Vector	Site of Gamogony and Sporogony	Locality	Authors
*Haemogregarina crocodilinorum*	*Alligator mississippiensis*	Intra-erythrocytic	*Placobdella multilineata*	Intestinal epithelial cells of the leech	Southern USA includes Arkansas, Carolina, and Florida	Börner [103]
*Haemogregarina caimani* *(= Hepatozoon caimani)*	*Caiman latirostris*	Intra-erythrocytic	*Culex dolosus*	Hemocoel	Brazil	Pessôa and de Biasi [104]
*Haemogregarina pettiti (=Hepatozoon pettiti Hoare 1932)*	*Crocodilus niloticus*	Erythrocytes and liver	*Glossina palpalis*	Intestine	Uganda, Senegal, West Africa	Hoare [105]
*Hepatozoon* sp.	*Caiman c. yacare*	Intra-erythrocytic	*Phaeotabanus fervens*	Intestine	Pantanal	Viana and Marques [106]
*Hepatozoon caimani*	*Caiman yacare*	Intra-erythrocytic	*--*	--	Pantanal region, Brazil	Viana et al. [107]

**Table 8:** Haemogregarines of birds.

Species of Haemogregarines	The Vertebrate Host	Site of Merogony	Invertebrate Vector	Site of Gamogony and Sporogony	Locality	Authors
*Hepatozoon atticorae*	*Hirundo spilodera*	Intra-erythrocytic	*Ornithodoros peringueyi* and *Xenopsylia trispinis*	Hemolymph	South Africa, South America, Jamaica, Europea	Bennett et al. [108]
*Hepatozoon prionopis*	*Prionops plumatus*	Intra-erythrocytic	--	--	Transvaal, South Africa	Bennett and Earle [109]
*Hepatozoon lanis*	*Lanius collaris*	Intra-erythrocytic	--	--	South Africa	Bennett et al. [108]
*Hepatozoon malacotinus*	*Dryoscopus cubla*	Intra-erythrocytic	--	--	South Africa	Bennett et al. [108]
*Hepatozoon numidis*	*Numida meleagris*	Intra-erythrocytic	--	--	South Africa	Bennett et al. [108]
*Hepatozoon pittae*	*Pitta arcuate*	Intra-erythrocytic	--	--	Sabah	Bennett et al. [108]
*Hepatozoon estrildus*	*Lonchura cucullata*	Intra-erythrocytic	--	--	Zambia	Bennett et al. [108]
*Hepatozoon sylvae*	*Parisoma subcaeruleum*	Intra-erythrocytic	--	--	South Africa	Bennett et al. [108]
*Hepatozoon zosteropis*	*Zosterops pallida*	Intra-erythrocytic	--	--	South Africa	Bennett et al. [108]
*Hepatozoon passeris*	*Sporopipes squamifrons*	Intra-erythrocytic	--	--	Botswana, South Africa	Bennett et al. [108]

**Table 9:** Haemogregarines of mammals.

Species of Haemogregarines	The Vertebrate Host	Site of Merogony	Invertebrate Vector	Site of Gamogony and Sporogony	Locality	Authors
*Hepatozoon perniciosum*	Laboratory white rats	The liver	*Echinolaelaps echidninus*	Stomach	Washington, USA	Miller [9]
*Hepatozoon griseisciuri*	*Sciurus carolinensis*	Bone marrow, liver, lung, and spleen (with intra-leucocytic gametocytes)	*Euhaemogamasus ambulans*, *Echinolaelaps echidninus* and *Haemogamasus reidi*	Stomach	Washington, Marland, Georgia, USA	Desser [110]
*Hepatozoon erhardovae*	*Clethrionomys glareolus*	Lung	*Xenopsylla cheopis*, *Ctenophthalmus agyrtes*, *C. assimilis* and *Nosopsyllus fasciatus*	Stomach and fat-body cells	Munich, Germany	Göbel and Krampitz [111]
*Hepatozoon sylvatici*	*Apodemus sylvaticus* and *Apodemus flavicollis*	Bone marrow and liver	*Laelaps agilis*	Stomach	Austria	Frank [112]
*Hepatozoon* sp.	Dogs	Intra-erythrocytic	--	--	Brazil	Forlano et al. [113]
*Hepatozoon canis*	Dogs	Intra-erythrocytic	--	--	Italy	Otranto et al. [114]
*Hepatozoon felis*	Cats	Intra-erythrocytic	--	--	India	Baneth et al. [115]
*Hepatozoon canis*	Dogs	Intra-erythrocytic	*Rhipicephalus sanguineus*	--	Mato Grosso do Sul, Brazil	Ramos et al. [116]
*Hepatozoon canis*	Dogs	Intra-erythrocytic	--	--	Central-western Brazil	Paiz et al. [117]
*Hepatozoon sp.*	*Cerdocyon thous, Nasua nasua, Leopardus pardalis, Canis familiaris, Thrichomys fosteri, Oecomys mamorae, Clyomys laticeps, Thylamys macrurus, Monodelphis domestics*	Intra-erythrocytic	*Amblyomma sculptum*, *A. parvum*, *A. tigrinum*, *Rhipicephalus microplus*, *R. sanguineus*, *A. auricularium*	--	Brazil	De Sousa et al. [118]
*Hepatozoon felis*	*Panthera leo*	--	*Rhipicephalus sanguineus*	--	Thailand	Bhusri et al. [119]
*Hepatozoon canis*	Dogs	Intra-erythrocytic	--	--	Czech Republic	Mitkova et al. [120]
*Hepatozoon felis*	Dogs	Intra-erythrocytic	*--*	--	Northeastern Iran	Barati and Razmi [121]
*Hepatozoon sp.*	Cats	Intra-erythrocytic	*--*	--	Turkey	Tuna et al. [122]
*Hepatozoon canis*	Dogs	Intra-erythrocytic	*--*	--	United Kingdom	Attipa et al. [123]
*Hepatozoon felis*	*Felis silvestris, Caracal caracal, Panthera pardus, P. leo, Leptailurus serval*	Muscle and Liver	*--*	--	Limpopo and Mpumalanga	Harris et al. [124]
*Hepatozoon luiperdjie*	*Panthera pardus*	Leukocytes	*--*	--	Limpopo Province, South Africa	Van As et al. [125]
*Hepatozoon canis*	*Dogs*	Intra-erythrocytic	*--*	--	Manila, Philippines	Baticados et al. [126]

**Table 10:** Primer sets used in the phylogenetic analysis of haemogregarines by *18S rRNA* gene.

Primer Set	Primer Sequence	Reference
4558F	5′- GCT AAT ACA TGA GCA AAA TCT CAA -3ʹ	Mathew et al. [146]
2733R	5′- CGG AAT TAA CCA GAC AAA T -3ʹ	
2867F	5′- AAC CTG GTT GAT CCT GCC AG -3′	Mathew et al. [146]
2868R	5′- TGA TCC TTC TGC AGG TTC ACC TAC -3′	
HEMO1	5′ - TAT TGG TTT TAA GAA CTA ATT TTA TGA TTG - 3′	Perkins and Keller [147]
HEMO2	5′ - CTT CTC CTT CCT TTA AGT GAT AAG GTT CAC - 3′	
HepF	5′- ATA-CAT-GAG-CAA-AAT-CTC-AAC -3′	Inokuma et al. [148]
HepR	5′- CTT-ATT-ATT-CCA-TGC-TGC-AG -3′	
HepF300	5′- GTTTCTGACCTATCAGCTTTCGAC -3ʹ	Ujvari et al. [20]
HepR900	5′- CAAATCTAAGAATTTCACCTCTGAC -3ʹ	
HEP-1	5′- CGC GAA ATT ACC CAA TT -3′	Criado-Fornelio et al. [149]
HEP-2	5′- CAG ACC GGT TAC TTT YAG CAG -3′	
Piroplasmid-F	5′- CCA GCA GCC GCG GTA ATT -3ʹ	Tabar et al. [150]
Piroplasmid-R	5′- CTT TCG CAG TAG TTY GTC TTT AAC AAA TCT -3ʹ	
EF	5′-GAA ACT GCG AAT GGC TCA TT-3′	Kvičerová et al. [26]
ER	5′-CTT GCG CCT ACT AGG CAT TC-3′	
Hep-001F	5′- CCT GGC TAT ACA TGA GCA AAA TCT CAA CTT -3′	Kledmanee et al. [151]
Hep-737R	5′- CCA ACT GTC CCT ATC AAT CAT TAA AGC -3′	
BTH-1F	5′- CCT GAG AAA CGG CTA CCA CAT CT -3′	Zintl et al. [152]
BTH-1R	5′- TTG CGA CCA TAC TCC CCC CA -3′	
GF2	5′- GTC TTG TAA TTG GAA TGA TGG -3′	Hodžić et al. [153]
GR2	5′- CCA AAG ACT TTG ATT TCT CTC -3′	
Haemog11_F	5′- ATT GGA GGG CAA GTC TGG TG -3ʹ	Rakhshandehroo et al. [99]
Haemog11_R	5′- GCG TTA GAC ACG CAA AGT CT -3ʹ	
HemoFN	5′- CCG TGG TAA TTC TAG AGC TAA TAC ATG AGC -3′	Alhaboubi et al. [100]
HemoRN	5′- GAT AAG GTT TAC GAA ACT TTC TAT ATT TA -3′	

**Table 11:** List of sequences for haemogregarines from GenBank database based on the *18S rRNA* gene.

Parasites	Hosts	Accession Number in GenBank
*Haemogregarina podocnemis*	*Podocnemis unifilis*	MF476203.1 - MF476205.1
*Haemogregarina pellegrini*	*Platysternon megacephalum*	KM887509.1
	*Malayemys subtrijuga*	KM887508.1
*Haemogregarina sacaliae*	*Sacalia quadriocellata*	KM887507.1
*Haemogregarina stepanowi*	*Emys orbicularis*	MT345287.1
	*Mauremys leprosa*	MT345284.1 - MT345286.1, KX691418.1, KX691417.1
	*Emys orbicularis*	KT749877.1, KF257928.1
	*Mauremys leprosa*	KF257929.1
	*Mauremys rivlata*	KF257927.1
	*Mauremys caspica*	KF257926.1, KF992697.1
*Haemogregarina bigemina*	*Lipophrys pholis*	MK393799.1 - MK393801.1
*Haemogregarina balli*	*Chelydra serpentine*	HQ224959.1
*Hepatozoon fitzsimonsi*	*Kinixys zombensis*	KR069084.1
	*Chersina angulate*	KJ702453.1
*Hepatozoon ursi*	*Ursus thibetanus japonicus*	EU041718.1, AB586028.1, LC431855.1 - LC431853.1
	*Melursus ursinus*	HQ829437.1 - HQ829429.1
*Hepatozoon seychellensis*	*Gradisonia alternans*	KF246566.1, KF246565.1,
*Hepatozoon ayorgbor*	*Apodemus sylvaticus*	KT274177.1, KT274178.1
	*Ctenophthalmus agyrtes*	KJ634066.1
	*Python regius*	EF157822.1
	*Rhombomys opimus*	MW342705.1
*Hepatozoon musa*	*Crotalus durissus*	MF497763.1 - MF497767.1
	*Philodryas natterei*	KX880079.1
*Hepatozoon involucrum*	*Hyperolius marmoratus*	MG041591.1 - MG041594.1
	*Ursus arctos*	MN150506.1 - MN150504.1
*Hepatozoon clamatae*	*Rana pipiens*	MN310689.1
*Hepatozoon catesbianae*	*Rana clamitans*	MN244529.1, MN244528.1, AF040972.1,
*Hepatozoon aegypti*	*Spalerosophis diadema*	MH198742.1
*Hepatozoon martis*	*Martes foina*	MG136688.1, MG136687.1
*Hepatozoon procyonis*	*Nasua nasua*	MF685386.1 - MF685409.1
*Hepatozoon griseisciuri*	*Scinurus carolinensis*	MK452389.1, MK452388.1, MK452253.1, MK452252.1,
*Hepatozoon sciuri*	*Scinus vulgaris*	MN104636.1 - MN104640.1,
*Hepatozoon americanum*	*Canis familiaris*	AF206668.1, KU729739.1
*Hepatozoon ingwe*	*Panthera pardus pardus*	MN793001.1, MN793000.1
*Hepatozoon theileri*	*Amietia quecketti*	KP119773.1, KX512804.1, KJ599676.1,
	*Amietia delalandii*	MG041605.1
*Hepatozoon caimani*	*Caiman crocodilus yacare*	MF322538.1, MF322539.1
	*Caiman crocodilus*	MF435046.1 - MF435049.1
*Hepatozoon silvestris*	*Felis silvestris silvestris*	KX757032.1
	*Felis catus*	MH078194.1, KY649445.1
*Hepatozoon tenuis*	*Afrixalus fornasini*	MG041595.1 - MG041599.1
*Hepatozoon thori*	*Hyperolius argus*	MG041600.1 - MG041603.1
*Hepatozoon ixoxo*	*Amietophrynus maculatus*	KP119772.1
*Hepatozoon luiperdjie*	*Panthera pardus pardus*	MN793002.1 - MN793004.1,
*Hepatozoon cuestensis*	*Crotalus durissus*	MF497769.1, MF497770.1
*Hepatozoon sipedon*	*Snakes*	AF110249.1 - AF110241.1
*Hepatozoon erhardovae*	*Megabothris turbidus*	KJ608372.1
*Hepatozoon domerguei*	*Furcifer* sp.	KM234649.1 - KM234646.1
*Hepatozoon tuatarae*	*Sphenodon punctatus*	GU385473.1 - GU385470.1
*Hepatozoon* cf. *ophisauri*	*Rhombomys opimus*	MW256822.1
*Hepatozoon colubri*	*--*	MN723844.1
*Hepatozoon canis*	*Amblyomma cajennense*	KT215377.1 - KT215353.1
	*Amblyomma sculptum*	KP167594.1
	*Tapir tapir*	MT458172.1
	*Haemaphysalis longicornis*	MT107092.1 - MT107097.1, MT107087.1 - MT107089.1, LC169075.1
	*Haemaphysalis concinna*	KC509532.1 - KC509527.1
	*Rhipicephalus sanguineus*	MH595911.1 - MH595892.1, MG807347.1, KY056823.1, MG241229.1, KT587790.1, KT587789.1, KY196999.1, KY197000.1 - KY197002.1, JQ867389.1, MN207197.1
	*Rhipicephalus microplus*	HQ605710.1
	*Rhipicephalus decoloratus*	MN294724.1
	*Canis lupus familiaris*	MH615003.1, EU289222.1, DQ071888.1, MK910141.1 - MK910144.1, MK757793.1 - MK757815.1, MN791089.1, MN791088.1, MN393913.1, MN393910.1, MK645971.1 - MK645946.1, MK214285.1 - MK214282.1, MG254613.1 - MG254622.1, MK091084.1 - MK091092.1, KY940658.1, MG772658.1, MG254573.1 - MG254611.1, KY021176.1 - KY021184.1, MG496257.1, MG496273.1, MG062866.1, MG076961.1, MG209580.1 - MG209594.1, KX588232.1, KU729737.1, KU729738.1, KY026191.1, KY026192.1, KX880502.1 - KX880506.1, KX761384.1, KU232309.1, KU232310.1, KT736298.1, LC012839.1 - LC012821.1, LC053450.1, JX976545.1, JN584478.1 - JN584475.1, JF459994.1, GQ176285.1, EU571737.1, EF650846.1, MW019643.1 - MW019630.1, MT909554.1, MT081051.1, MT081050.1, MT821184.1, MT499356.1 - MT499354.1, MT754266.1, LC556379.1, MT433126.1 - MT433121.1
	*Lycalopex vetulus*	AY150067.2, MT458173.1
	*Kinixys species*	MT704950.1
	*Lycalopex gymnocercus*	KX816958.1
	*Didelphis albiventris*	KY392884.1, KY392885.1
	*Canis aureus*	KF322145.1, KC886721.1, KC886729.1 - KC886733.1, KJ868814.1, KJ572977.1 - KJ572975.1, KJ634654.1, JX466886.1 - JX466880.1,
	*Felis catus*	KY469446.1, MN689671.1 - MN689661.1
	*Vulpes vulpes*	KF322141.1-KF322144.1, KC886720.1 - KC886728.1, MK757741.1 - MK757792.1, MN103520.1, MN103519.1, MH699884.1 - MH699892.1, MG077084.1 - MG077087.1, KY693670.1, KJ868819.1 - KJ868815.1, KU893118.1 - KU893127.1, KM096414.1 - KM096411.1, KJ572979.1, KJ572978.1, EU165370.1, GU376458.1 - GU376446.1, DQ869309.1, AY731062.1, MW295531.1, MN463026.1 - MN463021.1
	*Ixodes ricinus*	KU597235.1 - KU597242.1, KC584780.1
	*Hydrochoerus hydrochaeris*	KY965141.1 - KY965144.1
	*Cuon alpinus*	HQ829448.1 - HQ829438.1, MK144332.1
	*Dermacentor reticulatus*	KC584777.1 - KC584773.1
	*Pseudalopex gymnocercus*	AY471615.1, AY461376.1, AY461375.1
	*Panthera leo*	MT814748.1
	*Panthera tigris*	MT232064.1 - MT232062.1
	*Camelus dromedrius*	MN989311.1
*Hepatozoon apri*	*Sus scrofa leucomystax*	LC314791.1
	*Amietophrynus gutturalis*	KP119771.1
	*Amietophrynus garmani*	KP119770.1,
	*Sclerophrys maculata*	KX512803.1
	*Sclerophrys pusilla*	MG041604.1
*Hepatozoon* cf. *felis*	*Felis catus*	MK301457.1 - MK301462.1, MK724001.1, MG386482.1 - MG386484.1, KY649442.1 - KY649444.1, AY628681.1, AY620232.1
	*Felis silvestris silvestris*	KX757033.1, MT210593.1 - MT210598.1,
	*Puma concolor*	MT458171.1
	*Eira barbara*	MT458170.1
	*Lycalopex gymnocercus*	HQ020489.1
	*Leopardus pardalis*	KY684005.1
	*Asiatic lion*	KX017290.1
	*Prionailurus bengalensis*	AB771577.1 - AB771501.1, GQ377218.1 - GQ377216.1
	*Prionailurus iriomotensis*	AB636287.1 - AB636285.1
	*Panthera onca*	KU232302.1 - KU232308.1
	*Panthera tigris*	MT645336.1, MT634695.1
	*Rhipicephalus sanguineus*	JQ867388.1
	*Eurasian lynx*	MN905025.1, MN905023.1, MN905027.1
*Haemolivia parvula*	*Kinixys zombensis*	KR069083.1, KR069082.1
*Haemolivia stellata*	*Amblyomma dissimile*	MH196477.1 - MH196482.1, MH196475
	*Amblyomma rotundatum*	KP881349.1
*Haemolivia mariae*	*Egernia stokesii*	KF992712.1, KF992711.1
	*Tiliqua rugosa*	JN211118.1, HQ224961.1
*Haemolivia mauritanica*	*Hyalomma aegyptium*	MH618775.1, MN463032.1, MN463031.1, MW092781.1 - MW092776.1, MK918611.1 - MK918608.1, MH497199.1 - MH497190.1, MH975037.1, MH975031.1, MH975026.1, MH975025.1,
	*Hyalomma* sp.	MF383512. - MF383506.1,
*Haemolivia mauritanica*	*Canis lupus familiaris*	KP719092.1
	*Testudo marginata*	KF992710.1, KF992699.1
	*Testudo graeca*	KF992709.1 - KF992698.1, MH975039.1 - MH975032.1, MH975030.1 - MH975027.1, MH975024.1 - MH975021.1,
*Karyolysus paradoxa*	*Varanus albigularis*	KX011039.1, KX011040.1
*Karyolysus* cf. *lacazei*	*Ixodes ricinus*	MK497254.1

## Data Availability

All data generated or analysed during this study are included in this published article.
